# Reliable Assessment and Quantification of the Fluorescence-Labeled Antisense Oligonucleotides *In Vivo*

**DOI:** 10.1155/2014/196837

**Published:** 2014-05-25

**Authors:** Maria Chiara Munisso, Tetsuji Yamaoka

**Affiliations:** Department of Biomedical Engineering, National Cerebral and Cardiovascular Center Research Institute, Suita, Osaka 565-8565, Japan

## Abstract

The availability of fluorescent dyes and the advances in the optical systems for *in vivo* imaging have stimulated an increasing interest in developing new methodologies to study and quantify the biodistribution of labeled agents. However, despite these great achievements, we are facing significant challenges in determining if the observed fluorescence does correspond to the quantity of the dye in the tissues. In fact, although the far-red and near-infrared lights can propagate through several centimetres of tissue, they diffuse within a few millimetres as consequence of the elastic scattering of photons. In addition, when dye-labeled oligonucleotides form stable complex with cationic carriers, a large change in the fluorescence intensity of the dye is observed. Therefore, the measured fluorescence intensity is altered by the tissue heterogeneity and by the fluctuation of dye intensity. Hence, in this study a quantification strategy for fluorescence-labeled oligonucleotides was developed to solve these disadvantageous effects. Our results proved that upon efficient homogenization and dilution with chaotropic agents, such as guanidinium thiocyanate, it is possible to achieve a complete fluorescence intensity recovery. Furthermore, we demonstrated that this method has the advantage of good sensitivity and reproducibility, as well as easy handling of the tissue samples.

## 1. Introduction

Antisense oligonucleotides (ODNs) are very attractive tools for genetic-based therapies and treatments in modern medicine [1–4]. However, the cellular uptake of ODNs is poor due to their negatively charged backbone, degradation by nucleases, and uptake by nontarget cells. In response to these problems, cationic polymers and lipids, which spontaneously form complexes with the negatively charged ODNs, have been designed as delivery carriers. Complexes of ODNs and cationic polymers are internalized by endocytosis; then, an efficient route progresses with the endosomal escape, dissociation of complexes, and diffusion of ODNs in the cytoplasm and/or nucleus [5]. Actually, the main issues in developing appropriate delivery carriers are related with the knowledge on complexes incorporation, targeting, biodistribution, and localization within the organs and cells. Therefore, substantial efforts have been made to develop techniques capable of tracking the labeled ODNs pathway* in vivo* and to quantify the concentration in the tissues. Traditionally, tissue analysis was carried out using radiolabeled compounds [6], which are very accurate and sensitive, but currently their use has been limited because the safety issues are associated with radioactivity. Thus, alternative methods have been developed for quantification by optical absorption [7], fluorescence spectroscopy [8], and chromatography [9]. In particular, very useful method appears to be the optical imaging, which relies on the detection of photons produced by bioluminescence or fluorescence [10, 11]. Developments in fluorophore chemistries have resulted in a series of fluorophores with emissions extending from ultraviolet to near-infrared spectrum [12, 13]. In particular, near-infrared wavelengths are advantageous because tissue absorption and autofluorescence are minimized at these wavelengths. However, even in this range, it remains difficult to carry out an in-depth analysis of organs. In fact, only areas near the surface can be reliably detected [14–17]. In addition, although the fibrous and solid tissue samples are often homogenized prior to these analyses [18, 19] the determination of the fluorophores concentration is limited by the interplay of absorbers and scatterers in the tissue samples, which render tissue optically turbid. Thus, the observed fluorescence from biologic tissue is often significantly distorted and cannot be simply interpreted [20–25]. To overcome these problems, the fluorophores concentrations can be determined by fitting the tissue spectra to mathematical models [26], but accuracy and reliability can be achieved only when the effects of scattering and absorption are compensated by apposite correction techniques.

Furthermore, although it is well known that when the labeled ODNs bind cationic polymers its fluorescence is quenched [27, 28], these intensity changes have been neglected in almost all the presented analysis of data collected in* in vivo *experiments.

In this study, we proved the existence of fluorescence distortions due to the scattering/absorption of tissue samples and to the change in fluorescence emission of the fluorophores after complex formation. Moreover, we presented a method which avoids any misinterpretation of the fluorescence data and quantifies the concentration of the ODNs delivered by our liver specific carrier based on cationic diblock glycopolymer composed of galactosyl oxyethyl methacrylate (GAMA) and the primary amine-containing dimethylamino ethyl methacrylate (DMAEMA).

## 2. Materials and Methods

### 2.1. Materials

Dimethylamino ethyl methacrylate stabilized with MEHQ (DMAEMA, Mw 157.21. TCI Europe NV, Antwerp, Belgium) was used after purification. Glucosyl ureaethyl methacrylate (GUMA; Organic Chemical IND, LTD., Osaka, Japan) and galactosyl ureaethyl methacrylate (GAMA; Organic Chemical IND, LTD.),* N*-*N*-dimethylformamide dehydrate (DMF; Wako, Osaka, Japan), anhydrous dimethyl sulfoxide (DMSO; Wako, Osaka, Japan), tetrahydrofuran (THF; Wako, Osaka, Japan), 2-2′-bipyridyl (2-2′-bipyridine) (Bpy; Nacalai Tesque, Kyoto, Japan), copper (I) bromide (CuBr, 99.9%; Wako, Osaka, Japan), copper (I) chloride (CuCl, 99.9%; Wako, Osaka, Japan), ethyl 2-bromoisobutyrate (TCI Europe NV, Antwerp, Belgium), and methyl (±) *α*-bromophenylacetate (MBP, Fluka, Tokyo, Japan) were also used without further purification.

### 2.2. Antisense Oligonucleotides

The chemically modified 14-mer antisense phosphorothioate ODNs (proprotein convertase subtilisin kexin type 9 (PCSK9) antisense oligonucleotides) used in this study were synthesized by Gene Design (Osaka, Japan). The sequence used was as follows: 5′ CgTgggcagcagCC 3′; uppercase C and T indicate chemically modified 2′,4′-BNA/LNA that have a methylene bridge between the O2′ and C4′ atoms; lowercase indicates DNA [29, 30].

The ODNs-NH_2_ was labelled according to the protocol of the producers with the Alexa Fluor carboxylic acid succinimidyl ester (Alexa Fluor 750 and Alexa Fluor 594; Invitrogen, Carlsbad, CA) [31]. The dye conjugation to the ODNs was performed at pH 8.5 in 0.1 M sodium tetraborate buffer. The dye was dissolved at 1 mg/mL in DMSO and was immediately added to the ODNs solutions to obtain the desired dye-to-ODNs molar ratios while stirring. The reaction mixture was incubated overnight at room temperature. The conjugates were purified from unreacted dye by size-exclusion chromatography using Illustra MicroSpin G-25 Columns (GE Healthcare UK Limited). Absence of unreacted dye was assayed by thin-layer chromatography on silica gel plates using methanol: ethyl acetate (60 : 40).

### 2.3. Cationic Diblock Copolymers

**Figure 1:**
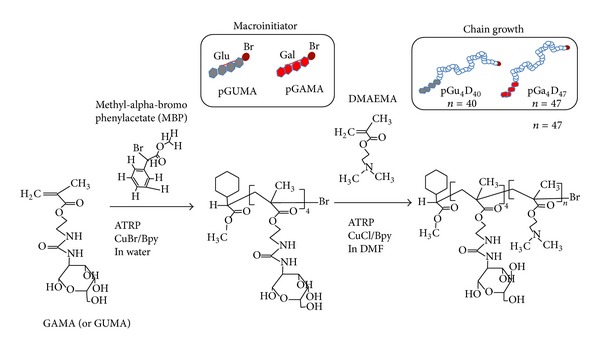
Schematic representation of the synthesis of pGu_4_D_40_ and pGa_4_D_47_.

Atom transfer radical polymerization (ATRP) has been employed for the polymerization of the two glycopolymers (pGa_4_D_47_ and pGu_4_D_40_) as previously described by the authors [32]. At first, a macroinitiator (pGAMA or pGUMA) was synthesized by ATRP in water and then used in a second ATRP reaction in DMF with DMAEMA monomer. We performed a two-step reaction in order to obtain a cationic diblock copolymer composed of a longer chain of pDMAEMA and a shorter chain of pGAMA (or pGUMA). We used the following nomenclature for the diblock polymers: pGu_*x*_D_*y*_ and pGa_*x*_D_*y*_, where *x* is the number of GAMA (pGa_*x*_) or GUMA (pGu_*x*_) monomeric units and* y *is the number of the DMAEMA monomeric units (Figure 1). The poly(dimethylamino ethyl methacrylate) (pDMAEMA) was synthesized by ATRP in THF using ethyl 2-bromoisobutyrate as initiator.

The copolymers were characterized by gel permeation chromatography (GPC) (Supplementary Figures: S. (1) in the Supplementary Material available online at http://dx.doi.org/10.1155/2014/196837) and ^1^HNMR measurements (Supplementary Figures S. (2), (3), and (4)). The GPC measurement was performed in a Shimadzu GPC system using a 0.5 M sodium acetate/0.5 M acetic acid buffer as eluent and a TSK Gel GMPWXL column (Tosoh Bioscience, Montgomeryville, PA) at room temperature and at a flow rate of 0.5 mL/min. PEI standards (Mw 400–890,000; Tosoh Bioscience, Montgomeryville, PA) were used for calibration. ^1^HNMR spectra were recorded on a Varian 300 MHz Instrument (Varian Inc., Tokyo, Japan) in D_2_O.

### 2.4. Preparation of Polyplexes

Copolymer/ODNs polyplexes differing in charge ratio but having the same ODNs concentration (56 nM) were prepared as follows. The polyplexes were prepared by adding different volumes of diblock polymer stock solution (buffer at pH 7.4) to a fixed volume of ODNs stock solution in one step. After addition of the polymer solution, the dispersion was vortexed for 10 sec. The polyplexes were allowed to equilibrate for 30 min at room temperature before use. The prepared polyplexes were electrophoresed on 1% agarose gels at 100 V for 20 min. After incubation for 20 min at room temperature, 10 mg/mL ethidium bromide solution was added and shortly incubated in the dark. Fluorescence was quantified using a Molecular Imager Gel Doc XR+ System (Bio Rad Laboratories, Inc.). In addition, dynamic light scattering measurements were carried out on a Malvern Zetasizer Nano ZS (Malvern, Worcestershire, UK) at 25°C and at an angle of 173 degrees. The incident beam was a HeNe laser beam (633 nm). Polystyrene nanospheres (60 ± 6 nm; Duke Scientific Corp, Palo Alto, CA) were used to verify the performance of the instrument. The particle size and zeta potential of each dispersion were measured three times. Furthermore, to examine the stability in acid environments, the polyplexes were diluted in aqueous citric acid/trisodium citrate buffer (pH 5.5). The polyplexes were incubated at room temperature for 24 hours and then were electrophoresed on 1% agarose gel.

### 2.5. *In Vivo* Experiment

#### 2.5.1. Fluorometry Measurement

Alexa 750-labeled ODNs were used to prepare polyplexes (N/P = 12) in HEPES-buffered glucose solution (20 mM HEPES, 5% glucose w/v, pH 7.4). 200 *μ*L of solution was administered to the C57/BL6 mice via the tail vein (6.3 *μ*g PCSK9 ODNs/mouse). After 3 and 48 hours mice were sacrificed and liver samples were harvested. Each liver was immersed in 2 mL of LRT solution (Lysis buffer solution, QuickGene RNA tissue kit SII, Fujifilm Corp.) and then mechanically homogenized (Digital Homogenizer and Tissue Grinder Glass Vessel with Teflon Pestle Homogenizer). Subsequently, aliquots of the resulting homogenate were diluted with additional LRT solution to reduce the optical density (OD) of the sample below 0.1 and stored for 24 hours. OD measurements were performed with UV1800 spectrophotometer (Shimadzu, Japan). Fluorometry measurements were performed in a scanning spectrofluorometer Thermo Scientific Varioskan Flash (Thermo Fisher scientific) and CRi's Maestro 500FL* in vivo *imaging system (Cambridge Research & Instrumentation, Incorporated).

#### 2.5.2. Confocal Microscopy Imaging

Alexa 594-labeled ODNs were used to prepare polyplexes (N/P = 12) in HBG. 200 *μ*L of solution was administered to the C57/BL6 mice via the tail vein (14 *μ*g PCSK9-ODNs/mouse). After 3 and 24 hours, mice were sacrificed and organs were embedded in Optimal Cutting Temperature compound (Tissue-Tek CRYO-OCT compound Fisher Scientific Inc.). Sections were cut and analyzed with Olympus IX81 (Olympus, Tokyo, Japan). Image sections were imported as 16 bit images and analyzed by NIH Image J software.

### 2.6. Ethic Statements

All animal experiments were conducted in accordance with the Guidelines for Animal Experiments established by the Ministry of Health, Labour and Welfare of Japan and by the National Cerebral and Cardiovascular Center Research Institute, Japan. The protocol was approved by the Committee on the Ethics of Animal Experiments of the National Cerebral and Cardiovascular Center Research Institute (Permit Number: 10047).

## 3. Results and Discussion

**Figure 2:**
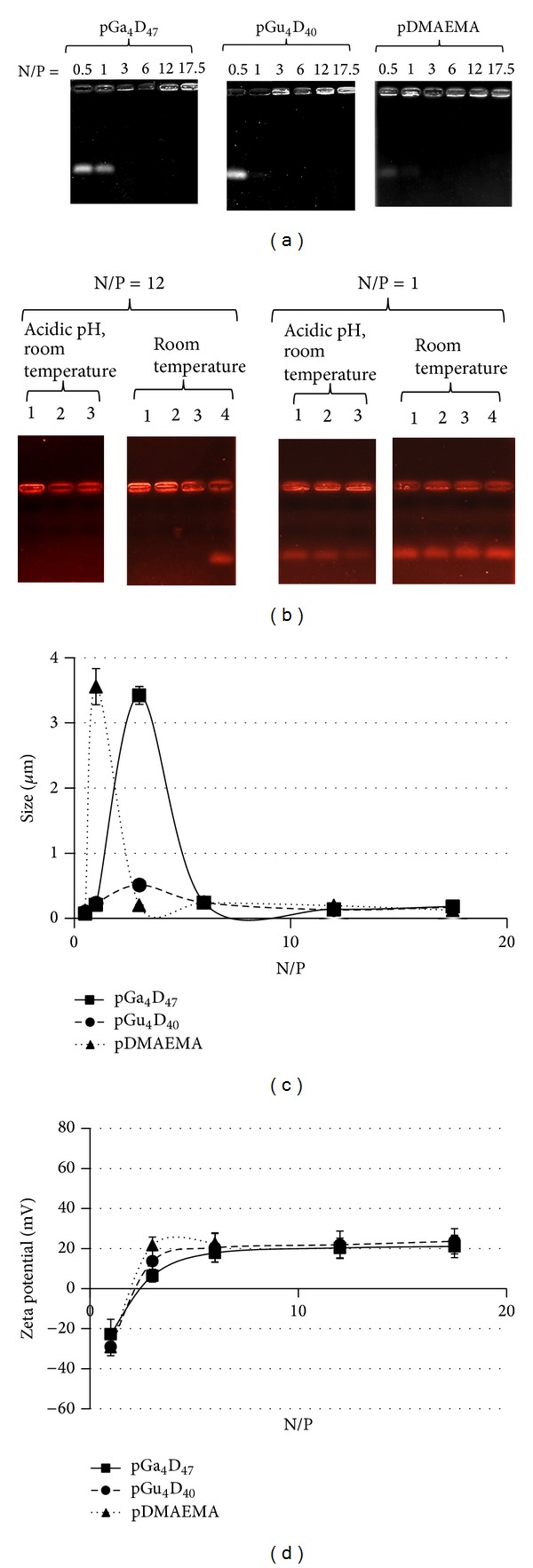
Gel retardation assays to evaluate (a) the stable polyplexes formation and (b) the binding strength of polyplexes in acidic pH. Line1: pGa_4_D_47_, line2: pGu_4_D_40_, line 3: pDMAEMA, and line 4: ODNs. (c) Size and (d) zeta potential of polyplexes (■) pGa_4_D_47_, (●) pGu_4_D_40_, and (▲) pDMAEMA.

Nowadays, it is of great interest to develop an efficient gene delivery to the hepatocytes, because these cells are responsible for the synthesis of a wide variety of proteins. Since hepatocytes possess a large number of asialoglycoprotein receptors, specific targeting has been achieved by using ligand bearing galactose units [33, 34]. For this reason, two diblock glycopolymers and a copolymer of DMAEMA were synthesized by ATRP (Figure 1). The molecular weight of pDMAEMA was estimated to be around 8000 g/mol as determined by GPC and ^1^HNMR and the total content of nitrogen was 6.3 *μ*mol/mg polymers. The two glycopolymers were synthesized by consecutive ATRP reactions, as previously described [32]. The total content of nitrogen was 5.4 and 5.3 *μ*mol/mg polymers, for pGa_4_D_47_ and pGu_4_D_40_, respectively. The carrier ability to form stable polyplexes with ODNs at N/P ratios greater than 1 was analyzed with gel retardation assay (Figure 2(a)). The influence of sugar units on polyplexes formation was very weak. The binding strength of the polyplexes at N/P ratio of 1 and 12 was also examined in response to acidic pH (Figure 2(b)). After incubation for 24 hours at pH 5.5, the polyplexes prepared at N/P ratio of 12 did show large binding strength and ODNs were not released from the polyplexes. Instead, in polyplexes prepared at N/P ratio of 1, almost all the ODNs were released even at room temperature. However, it is important to notice the positive effect of the acid pH on the binding strength of these polyplexes. In fact, after 24 hours of incubation at pH 5.5, the quantity of released ODNs decreased. Indeed, under acidic conditions the increase in the positive charge of protonated pDMAEMA blocks leads to a rise of the binding strength between carriers (positive charge) and ODNs (negative charge). This effect is higher for pDMAEMA (Figure 2(b)), which had shown the larger binding strength also at lower N/P (Figure 2(a)).

The size and zeta potential of polyplexes of all the carriers were examined at N/P ratios from 1 to 17 (Figures 2(c) and 2(d)). The hydrodynamic diameter varied over a wide range (100–3500 nm) at N/P ratios smaller than 6 in all the copolymers (Figure 2(c)). At N/P ratio of 12, the diameter was smaller than 200 nm (Figure 2(c)) and was slightly influenced by the different sugar units.

**Figure 3:**
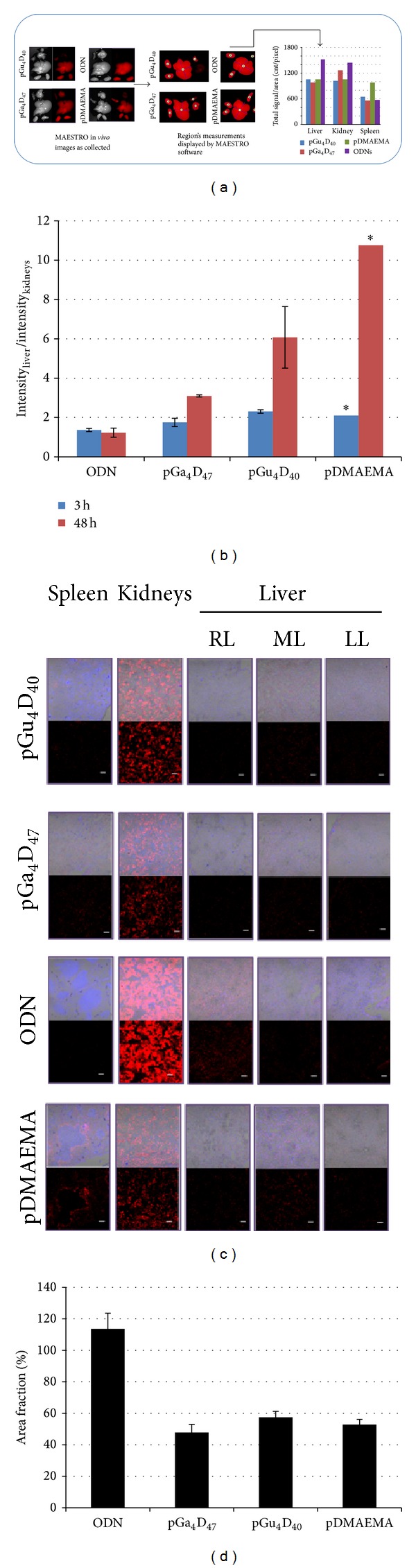
(a) Schematic representation of the experimental procedure. (b) Ratio of the total emission from liver and kidneys (*N* = 2, ∗ = 1). (c)* In vivo* organ imaging using confocal microscope. Scale 100 *μ*m. ML = median lobe, RL = right lateral and caudal lobe, and LL = left lateral lobe. The organs were obtained from mice at 3 hours after injection via tail vein. (d) Area fraction of antisense-Alexa 594 in mice sacrificed after 3 hours.

In the* in vivo* experiments (Figure 3) N/P ratio of 12 was used. The images were collected by MAESTRO* in vivo* imaging systems (Figures 3(a) and 3(b)) and by confocal microscopy (Figures 3(c) and 3(d)). MAESTRO imaging system allows unmixing the dye spectrum from the autofluorescence and performing quantitative image analysis (Figure 3(a)). To avoid systematic errors, I previously determined the range of linearity of the detection system by the variation of the light intensity via fluorophore concentration [35]. In addition, to avoid many method-inherent problems connected with the comparison of fluorescence intensities collected from different samples (e.g., instrument-specific wavelength and polarization dependences), the internal relative fluorescence emission ratio between the liver and kidneys was evaluated (Figure 3(b)). A time dependent increase in the emission ratio was observed for glycopolymers and pDMAEMA. Instead, free ODNs injection showed no detectable change. However, it is not possible to explain such a large increase in the emission ratio considering only the different blood circulation time of ODNs and polyplexes. Therefore, a more detailed analysis on the labeled ODNs distribution was performed on cryosections by confocal microscopy (Figure 3(c)). The higher fluorophore emission is detected in the kidneys of mice treated with free ODNs. This confirms previous work which reported that although the ODNs are accumulated particularly in the liver, high accumulation can be detected in the kidneys, their primary route of elimination [36]. The total emission per animal (Figure 3(d)) was determined by Image J software as the sum of the partial area fraction associated with each organ emission (Figure 3(c)). It is possible to observe that the higher total emission is collected in animals treated with free ODNs (Figure 3(d)). Since the total quantity of labeled ODNs is the same in all the animals, this discrepancy can be explained only assuming that the fluorescent dye emission changes after complexation with the carrier. Therefore, to confirm our hypothesis we decided to study the fluorescence stability of the carrier-free labeled ODNs and polyplexes.

**Figure 4:**
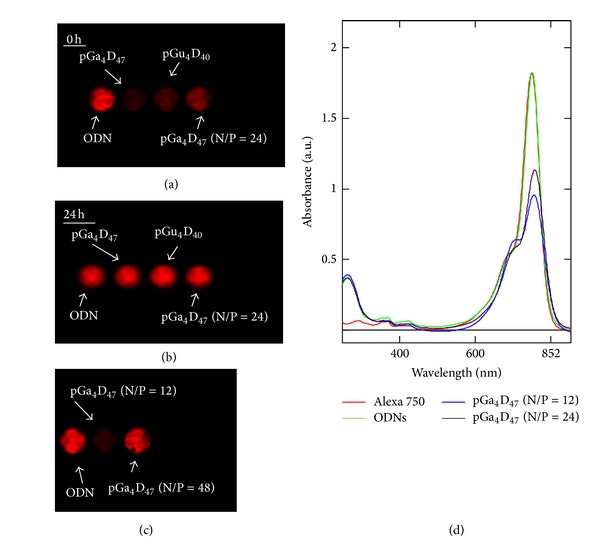
Change in emission as collected by Cri MAESTRO 500 FL imaging system. (a) Polyplexes and ODNs just prepared, (b) after 24 hours, and (c) ODNs and pGa_4_D_47_ complexes just prepared at N/P ratio of 12 and 48. (d) Absorbance spectra of Alexa 750, free labeled ODNs, and labeled ODNs after complexation with pGa_4_D_47_ at different N/P ratio.

The emissions of samples containing the same quantity of ODNs-labeled Alexa 750 were collected by MAESTRO and reported in Figures 4(a), 4(b), and 4(c). An evident fluorescence intensity reduction was observed in the polyplexes samples (Figure 4(a)). This is confirmed by the work of van Rompaey et al. [28], which reported that the presence of several rhodamine labeled ODNs chains in one complex can decrease the fluorescence emission on complexation with pDMAEMA [28]. In addition, the authors of the paper reported that the fluorescence emitted by labeled ODNs after complexation at different N/P ratio has a minimum at low N/P ratio and after the fluorescence increases again with the increase of N/P ratio, as consequence of the decrease of ODNs labeled molecules per complex, as confirmed by the emissions of pGa_4_D_47_ at N/P ratio of 12 and 48 and at N/P ratio of 24 reported in Figures 4(c) and 4(a).

Furthermore, we used absorption spectroscopy to look for evidence of dye-ODNs-carrier interactions and to determine the possible reasons for this difference in fluorescence emissions. In Figure 4(d) the Alexa 750, free labeled ODNs, and labeled ODNs in the complex with pGa_4_D_47_ spectra are shown. The absence of change in the absorbance around 260 nm suggests that the presence of carrier does not perturb the interaction of dye and ODNs. Instead, the increase in the absorbance of the shoulder at 700 nm and the decrease at 750 nm imply that the carrier interacts somehow with the dyes. In particular, we observed a much prominent shoulder peak at N/P ratio of 12 compared with N/P ratio of 24. In our opinion, at N/P ratio of 12 the tightly compacted structure of the polyplexes might lead to a larger fluorescence quenching due to dye-dye interactions [27, 28, 37], which are characterized by a decrease in the absorption band and an increase in a nonfluorescent shifted band.

Therefore, the reorganization of the polyplexes is at the origin of the curve of the fluorescence emission of the dye. At the minimum of fluorescence, a large number of fluorescence labeled molecules are entrapped in one polyplex, quenching each other due to their close spatial proximity. These considerations might justify the discrepancy in the total emission reported in Figure 3(d) and suggest that experimental fluorescence data are heavily affected by this quenching.

**Figure 5:**
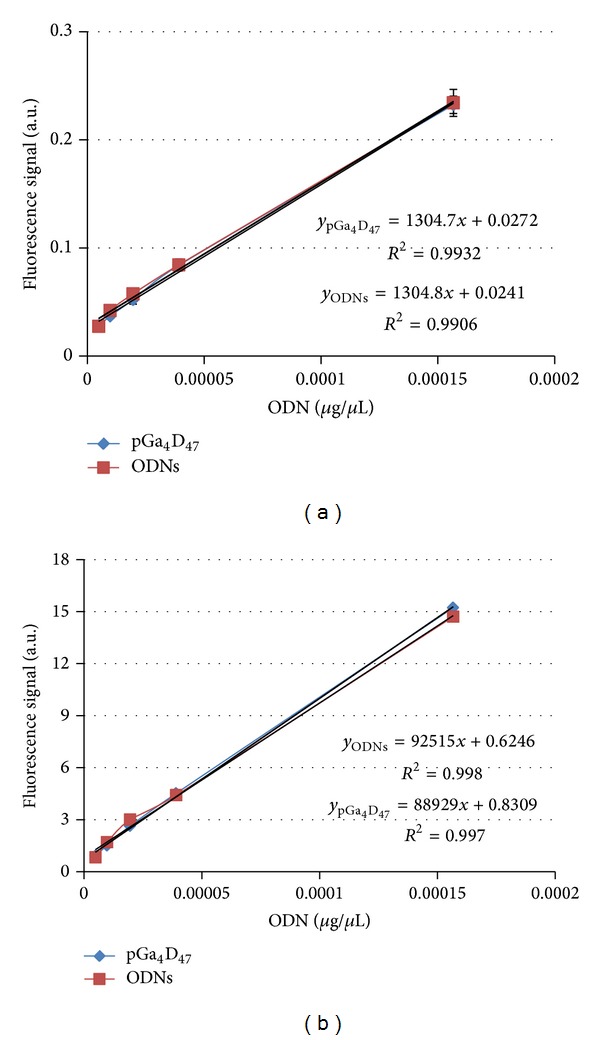
Standard curve emission as collected by Cri MAESTRO 500 FL imaging system (a) and Varioskan (b).

Finally, to define the fluorophore concentration inside the organs it is necessary to reduce common processes, such as absorption and scattering. In nonabsorbing media the light can penetrate deep into the sample and can be emitted isotropically; thus the fluorescence spectrum can be expressed as a linear combination of the fluorescence contributions of all N fluorophores in the sample. However, in turbid media, such as biological tissue, the fluorescence spectrum is not only dependent on the concentrations of fluorophores. In fact, at microscopic level, tissue can be considered as an absorbing bulk material with scatterers randomly distributed [38]. As consequence, some parts of the light will possibly go out after multiple scattering and some will be absorbed. This absorbed light will be converted to fluorescence and will continue to scatter in the media, where it can either be reabsorbed or be emitted from the sample surface. Thus, in absorbing media it is necessary to model the effects of the fluorophores, absorbers, and scatterers [39, 40]. However, the optical properties of these groups depend on used wavelengths, tissue types, and penetration depth of the light in tissues. Therefore, homogenization and dilution of the samples to achieve OD < 0.1 are necessary [25]. In fact, only for very dilute solution the measured fluorescence intensity *I* is proportional to the fluorophore's absorptance (fraction of radiation absorbed at specific wavelength) and not to absorbance (logarithmic ratio of the radiation falling upon a material to the radiation transmitted through a material). This ensured that the samples are uniformly excited during fluorometry and the light is emitted almost isotropically. At these conditions, the dye concentration [ODNs] in the tissue can be calculated as follows:

(1) $$\begin{array}{c} {\left[\text{ODNs}\right]}_{\text{liver}}=\frac{1}{\alpha }D_{1}D_{2}, \end{array}$$

where the last two factors account for the dilution of the tissue samples, *D*_1_ mixing the solid sample with 2 mL LRT, and *D*_2_ the final adjustment of the homogenized sample to the OD required. The fluorometer responsivity (*α*) was determined by linear regression fits of the emitted signal as a function of the known dye concentration in solution. The response was linear over the concentration range tested, with a correlation coefficient *R* ^2^ > 0.99 (Figure 5 and S.5). However, this method with double dilution steps cannot affect the number of labelled ODNs that are present in one polyplex, quenching each other due to their close spatial proximity (Figure 4(a)). Therefore, we hypothesized that the disruption of the polyplexes might determine a recovery of the fluorescence intensity.

It is well known that chaotropic agents can disrupt the structure of the macromolecules, such as proteins and nucleic acids by interfering with noncovalent interactions [41–43]. We think that guanidine thiocyanate can modify the hydrophilic/hydrophobic balance [25] and compromise the polyplex stability. This inevitably results in the polyplexes disruption. For this reason, we added the LRT solution (main component is guanidine thiocyanate) to the homogenized samples till OD < 0.1 and proved that the complete intensity recovery can be achieved in 24 hours (Figure 4(b)). In particular, we do not observe any change in the fluorescence intensity of the free labelled ODNs before or after 24 hours. This might suggest that the change in the fluorescence emission is connected only with the dissolution of the polyplexes, as previously hypothesized. Therefore, the liver samples were first homogenized in LRT and then diluted with the same solution till OD < 0.1 after 24 hours of incubation samples were analyzed by MAESTRO and Varioskan.

**Figure 6:**
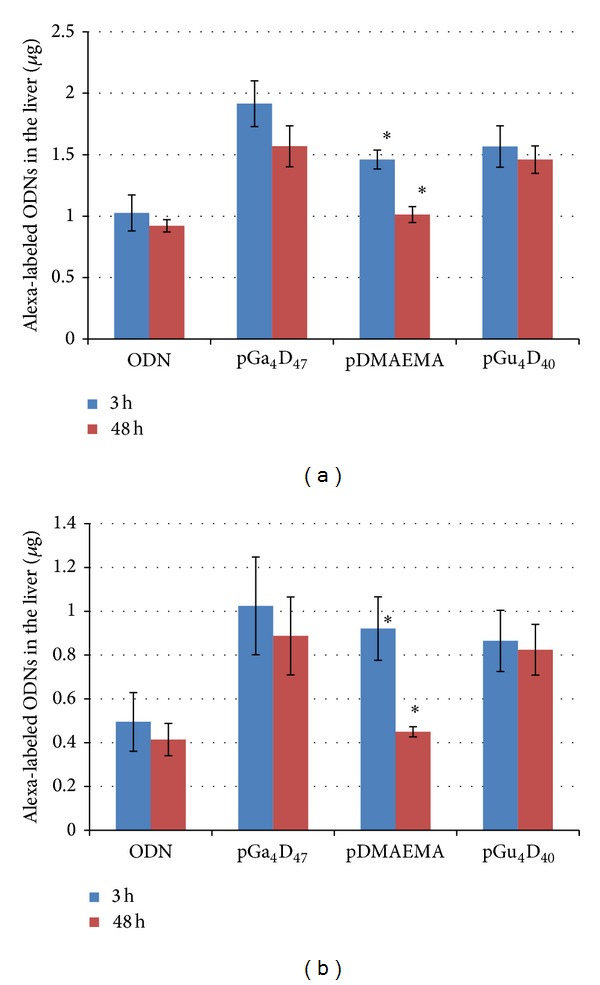
Quantification of the ODN accumulated in the liver by (a) MAESTRO and by (b) Varioskan. *N* = 2, ∗ = 1.

The quantification results are reported in Figure 6. About 17% of the original ODNs injected could reach the liver. Instead when pGa_4_D_47_ was used as carrier about 32% of the ODNs reach the liver within 3 hours. This carrier could deliver almost double quantity ODNs with respect to the injection of free ODNs. In addition, the quantity of ODNs delivered by the carrier bearing the galactose moieties is higher than the carrier bearing the glucose unit (about 24%) and the pDMAEMA (about 24%) (Figure 6(a)). Similar results were obtained when the fluorescence was measured by Varioskan. The difference in the experimental results is connected with the experimental errors. In fact, comparing the pGa_4_D_47_/ODN, pGa_4_D_47_/pDMAEMA, and pGa_4_D_47_/pGu_4_D_40_ ratios obtained at 3 h and 48 h with the two machines (data not shown), the range of errors is about 10–20%. Since it has been reported that if no polarizers are employed, the measurement uncertainty is in the range of ca. 20% [44], our method has a good degree of repeatability. Moreover, it has the advantage of an adequate detection limit, which depends on the fluorophores extinction coefficient, quantum yield, and also the tissue pigmentation. In particular, we proved that this assessment and quantification method is sensitive also in highly pigmented tissues, such as liver, which required higher dilution to reach the OD < 0.1.

## 4. Conclusion

Despite the widespread use of fluorescence techniques to quantify the accumulated labeled ODNs, many method-inherent problems (e.g., different absorption/scattering of different tissues and change in the absorption/emission spectra of labelled ODNs bind to carriers) and their influence on quality and reliability of measurements are still often neglected. In this paper a quantification method, which is independent from the tissues and fluorophores types, was presented and analyzed. This method is based on (i) a preliminary study of the fluorescence emission change of labelled ODNs on complexation with carriers, (ii) dilution of the tissue samples to achieve OD < 0.1 at each wavelength used, and (iii) a longer solubilisation time in solvent containing chaotropic agents to completely recover the original dye emission. This method has the advantages of sensitivity and reproducibility. In particular, the detection limit allows experiment with small tissue samples and low dye concentrations; therefore it will be adequate for clinical and preclinical studies.

## Supplementary Material

Gel permeation chromatography (GPC) results of the macro-initiators and co-polymers are reported in S.(1). 1H NMR measurements of MBP (S.(2)A), pGAMA (S.(2)B), DMAEMA (S.(2)C), pGa4D47 (S.(3)) and pGu4D40(S.(4)) are reported in S. (2), (3), and (4) respectively. In S.(5) are reported the tabulate of the fluorescence data of the Standard curve by (A) MAESTRO and (B) Varioskan. The curves are plotted in Figure: 5.
